# The Validation of a Single Multiplex Typing System With 45 Y-STR Markers for Familial Searching and Database Construction

**DOI:** 10.3389/fgene.2022.842004

**Published:** 2022-01-27

**Authors:** Ying Zeng, Ling Chen, Mengge Wang, Chengliang Yang, Hong Liu, Cheng Xiao, ChangHui Liu, Yue Li, Quyi Xu, Weian Du, Chao Liu

**Affiliations:** ^1^ School of Forensic Medicine, Southern Medical University, Guangzhou, China; ^2^ Guangzhou Forensic Science Institute, Guangzhou, China; ^3^ Faculty of Forensic Medicine, Zhongshan School of Medicine, Sun Yat-Sen University, Guangzhou, China; ^4^ Guangdong Homy Genetics Incorporation, Foshan, China

**Keywords:** developmental validation, forensic genetics, Y45 kit, slowly and moderately mutating Y-STR, database construction

## Abstract

The Y-chromosomal short tandem repeat (Y-STR) is an effective forensic tool in familial searches and patrilineal relationship evaluation. However, currently available Y-STR panels often lack sufficient discriminatory power to resolve genetic relationships between distant relatives or within patrilocal populations. This study aims to establish a novel Y-STR amplification system for forensic casework analysis and database construction, which contains 44 slowly and moderately mutating and one rapidly mutating Y-STR. The validation of the assay was conducted following the recommendations of SWGDAM developmental validation guidelines. Different types of casework samples were tested and reliable profiles were obtained. Furthermore, we genotyped and analyzed 141 unrelated Han Chinese male samples. The results showed that this Y45 kit could improve the performance of identifying male individuals, higher haplotype diversity, and discrimination capacity when compared to the previous widely used Yfiler Plus kit. In general, the validation study demonstrated that the newly developed Y45 kit possesses high sensitivity, inhibitor tolerance, male specificity in a mixture, species specificity, and precision and is capable of forensic casework analysis and database construction.

## 1 Introduction

Inherited DNA polymorphisms located in the non-recombining portion of the human Y chromosome (NRY) provide a powerful tool for tracking the patrilineal ancestry of male individuals (Poznik et al., 2016; Jobling and Tyler-Smith, 2017; Kayser, 2017; Khan et al., 2017). Various kinds of markers on the Y chromosome, especially the Y-chromosomal short tandem repeats (Y-STRs), have been widely utilized in forensic genetics, particularly in cases where standard autosomal DNA profiling is not informative (Yao et al., 2016; Kayser, 2017; Khan et al., 2017). The Y chromosome is passed from the father to the son unchangeably without considering the gradual accumulation of mutations (Jobling et al., 1997; Jobling and Tyler-Smith, 2017); thus, male individuals in the same paternal lineage share identical Y-STR haplotypes. In the last few decades, the Y-STR database has been set up to characterize paternal lineages of unknown male trace donors and acquired great achievements (Kayser, 2017). Generally, the mutation rates and discriminatory capacity (DC) of a Y-STR amplification system are two key forensic parameters to measure its practicability. Previous studies have shown that Y-STRs could be classified into three groups based on mutation rates: slowly mutating (SM, < 1.0 × 10^−4^), moderately mutating (MM, 1.0 × 10^−4^ ∼ 1.0 × 10^−2^), and rapidly mutating (RM, > 1.0 × 10^−2^) (Ballantyne et al., 2010; Ballantyne et al., 2014; Kayser and Ralf, 2018).

Currently, the common commercial Y-STR kits mainly include MM and RM Y-STRs. A previous study indicated that added RM Y-STRs would improve the efficiency of the system (Ballantyne et al., 2014) but mistakenly exclude male individuals from the same familial lineages, which could increase the difficulties of conducting paternal searching in the Y-STR database (Kayser, 2017). The SM Y-STRs are less likely to mutate among male relatives in the same pedigree, which makes this marker more suitable for forensic pedigree searches (Kayser, 2017; Liu et al., 2021). Therefore, we attempt to design a novel Y-STR panel with relatively low mutation rates of Y-STRs.

In this study, we constructed a new 45-plex Y-STR typing system, including 21 Y-STRs from a Yfiler Plus system (Applied Biosystems, Foster City, CA, United States) and 24 Y-STR loci with low to moderate mutation rates. Various tests were performed to evaluate the efficiency of the system, including PCR condition study, sensitivity, inhibitor study, mixture, species specificity, and stutter calculation. The developmental validation was conducted following the guidelines issued by SWGDAM (Daniels et al., 2004).

## 2 Materials and Methods

### 2.1 Sample Collection and Extraction

This study was approved by the Ethics Committee of Southern Medical University, and all the procedures were carried out following the recommendations of the Declaration of Helsinki (Nicogossian et al., 2014). Control DNA 2800M was purchased from Promega (Promega Corporation, Madison, WI, United States). Control DNA 9948 was provided by AGCU ScienTech Incorporation (Wuxi, China). Male chimpanzee oral swab was donated by Guangzhou Zoo (Guangzhou, China), and blood samples of eight male animal species (dog, cat, pig, rabbit, chick, duck, rat, and cow) were accumulated from Guangzhou Forensic Science Institute over years. Furthermore, 30 casework samples were selected from Guangzhou Forensic Science Institute, and 141 unrelated male blood samples were collected with informed consent. All genomic DNA was extracted using the Bokun magnetic bead kit (Bokun Biotech, Changchun, China) using the Freedom evo-150 base DNA system (TECAN, Hombrechtikon, Switzerland) following the manufacturer’s protocol. The samples were quantified using the Applied Biosystems^®^ QuantStudio^™^ 7 Flex Real-Time PCR System following guidelines as recommended by the manufacturer and diluted to approximately 2 ng/μL with TE buffer.

### 2.2 STR Loci Selection

**FIGURE 1:**
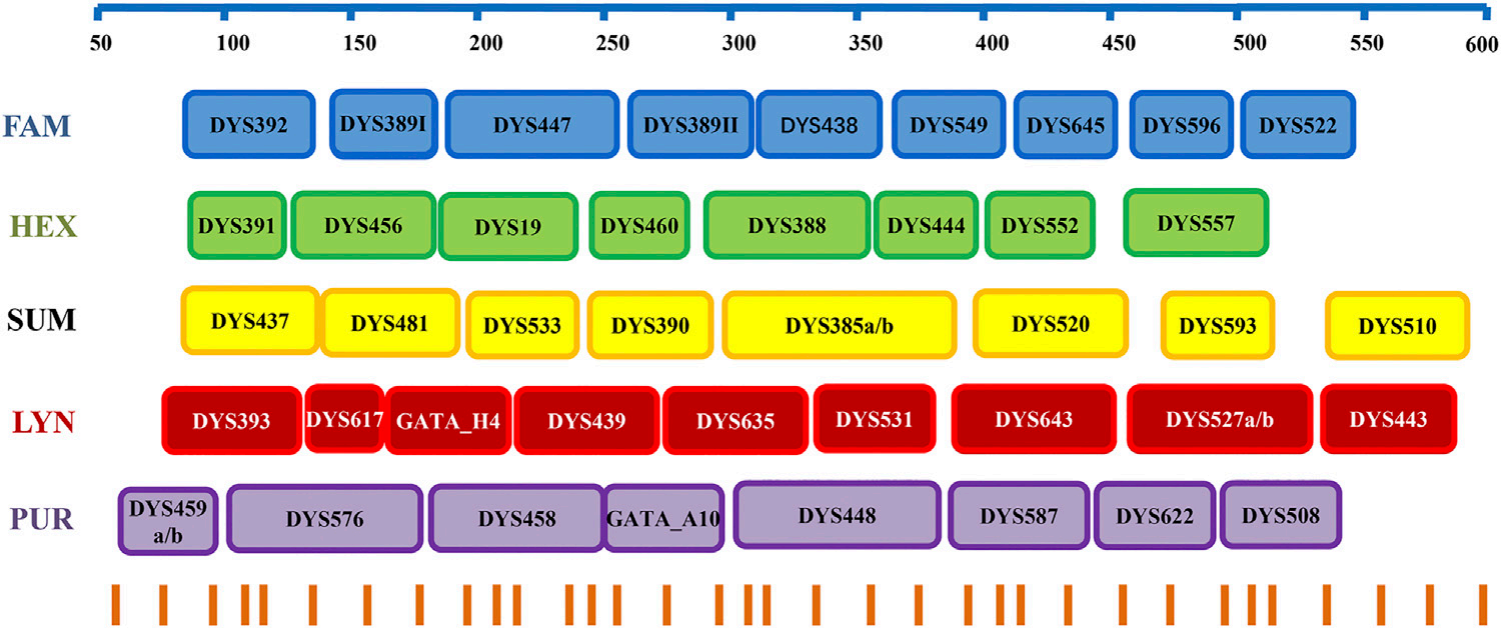
Diagram of the Y45 kit with five fluorescent dyes.

The Y-STR loci with slow and moderate mutation rates were selected based on the studies of Ballantyne et al. (2010), Ballantyne et al. (2014), and Kayser and Ralf (2018). Subsequently, a Y45 kit including DYS392, DYS389I/II, DYS438, DYS391, DYS456, DYS19, DYS460, DYS437, DYS481, DYS533, DYS390, DYS385a/b, DYS393, Y-GATA-H4, DYS439, DYS635, DYS458, DYS448, DYS447, DYS549, DYS645, DYS596, DYS522, DYS388, DYS444, DYS552, DYS557, DYS520, DYS593, DYS510, DYS617, DYS531, DYS643, DYS527a/b, DYS443, DYS459a/b, Y-GATA-A10, DYS587, DYS622, and DYS508 (Supplementary Table S1) was designed by AGCU ScienTech Incorporation (Wuxi, China). Five fluorescent dyes (FAM, HEX, SUM, LYN, and PUR) were used to label the primers (Figure 1), and fluorescent dye SIZ was employed as the internal lane standard. A total of 21 loci (DYS392, DYS389I/II, DYS438, DYS391, DYS456, DYS19, DYS460, DYS437, DYS481, DYS533, DYS390, DYS385a/b, DYS393, Y-GATA-H4, DYS439, DYS635, DYS458, DYS448, and DYS576) from the Yfiler Plus system (Applied Biosystems, Foster City, CA, United States) were included to meet the compatibility of the existing database, and other twenty-four loci were further proved to be polymorphic in Han Chinese with low and moderate mutation rates (Fan et al., 2021).

### 2.3 PCR Amplification, Electrophoresis, and Data Analysis

DNA samples were amplified on a GeneAmp^®^ PCR System 9700 thermal cycler (Thermo Fisher Scientific). Each amplification reaction contained 4 μL premix, 2 μL primer, 0.1–2 ng template DNA or 1.2 mm punch of the FTA card sample, and sdH_2_O to obtain a final reaction volume of 10 μL. Standard thermal cycling was performed under the following conditions: initial denaturation at 95°C for 10 min, 30 cycles of 94°C for 30 s, 60°C for 1 min, and 66°C for 1 min, followed by a final extension at 60°C for 20 min. Electrophoresis was performed on the Applied Biosystems 3500xl Genetic Analyzer (Thermo Fisher Scientific) and analyzed with GeneMapper^®^ ID-X Software v1.6.

### 2.4 PCR Condition Study

To validate the recommended PCR parameters of the user’s manual and optimize the PCR amplification condition, the cycling number, annealing temperature, and final extension time were tested. For each study, 0.5 ng of control DNA 9948 was prepared in triplicate, with 4 μL reaction mix, 2 μL Y45 primers, 0.4 μL C-Taq, and sdH_2_O to fill the total volume to 10 μL.

For the cycle number test, the system was amplified in series of 28, 29, 30, 31, and 32 cycles; the recommended thermal cycling number is 30.

For the annealing temperature test, the temperature was designed in increments of 59, 61, 63, 65, and 67°C; 63°C is the recommended temperature in the protocol.

For the final extension test, the final time was respectively held in the gradient of 10, 15, 20, 25, and 30 min; 15 min is the recommended time.

### 2.5 Sensitivity

To evaluate the sensitivity of the Y45 kit, the male control DNA 9948 was serially diluted with TE buffer to 1.0, 0.5, 0.25, 0.125, 0.0625, and 0.03125 ng each 1 μL.

### 2.6 Inhibitor Study

Four common inhibitors, namely, hemoglobin, indigo, humic acid, and EDTA were employed to assess the stability of the Y45 kit. The mixture template contained constant 0.5 ng male control DNA 9948 with inhibitors at following concentrations: 100, 200, 400, 600, 800, and 1,000 μmol/L of hemoglobin; 2, 8, 16, 20, 24, and 30 mmol/L of indigo; 10, 20, 40, 60, 80, and 100 ng/μL of humic acid; and 0.2, 0.4, 0.8, 1.0, 1.2, and 1.5 mmol/L of EDTA. The tests of all concentrations were performed three times.

### 2.7 DNA Mixtures

Male/male DNA mixtures were prepared with control DNA 9948 and 2800M, and the mixture ratios varied at 1:1, 1:3, 1:9, and 1:19. Female/male DNA mixtures were tested with control DNA 9947A and 9948 at 1,000:1, 100:1, 10:1, and 1:1 ratios. Each of the samples above was tested with a total DNA template of 0.5 ng and amplified in triplicate.

### 2.8 Species Specificity

Several non-human genomic DNA samples, including dog, cat, pig, rabbit, chick, duck, rat, and chimpanzee, were tested for cross-reactivity. The quantity of the DNA samples mentioned before was permanently held at 0.5 ng.

### 2.9 Reproducibility

A total of 50 samples accumulated from daily work were prepared for the reproducibility study. Three laboratories (Guangzhou Forensic Institute and two of its affiliated institutes) were employed to genotype the same DNA samples, and the generated results were compared to estimate the reproducibility.

### 2.10 Stutter Calculation

A 24-injection run of the AGCU Y45 allelic ladder with internal ladder standard (ILS) was performed on a 3500xl Genetic Analyzer. The AGCU Marker SIZE-600 size standard and GeneMapper^®^ ID-X Software v1.6 were used to conduct the standard deviation calculation on the observed allelic sizes. To determine the stutter ratios, 50 samples from the population study were randomly selected to calculate the stutter percentage by dividing the height of the stutter peak by the main allele peak height.

### 2.11 Casework Samples

A total of 30 common casework samples collected from the daily work were tested aiming to access the efficiency of the kit. These samples included muscle tissue (two), old bone (three), hair (five), nail (four), bloodstain (six), semen (two), teeth (three), saliva on cigarette (three), swabs of bottles (one), and swabs of ropes (one).

### 2.12 Population and Concordance Study

To conduct the population study, 141 unrelated healthy Han Chinese males were genotyped using the Y45 kit with informed consent. Additionally, all 141 male samples had been genotyped by Yfiler Plus (Applied Biosystems, Foster City, CA, United States) beforehand, and the concordance was evaluated by comparing the alleles obtained by these two kits. Relevant forensic parameters such as the haplotype diversity (HD), match probability (MP), and discrimination capacity (DC) were calculated.

## 3 Results and Discussion

### 3.1 PCR Condition Study

In this study, each reaction mixture consisted of 4 μL reaction mix, 2 μL Y45 primers, 0.4 μL C-Taq, and sdH_2_O to fill the total volume to 10 μL. The amount of DNA template was 0.5 ng, and all experiments were repeated three times.

#### 3.1.1 Cycle Number

The full profiles for all replicates were obtained at different cycle numbers (28–32 cycles). The corresponding average peak heights were 1792, 4,068, 5,826, 9776, and 17147, respectively. An expected increase of peak height has occurred under the additional cycles. The cycle numbers had no significant influence on the color balance. Off-scale peaks were detected at the 32^nd^ cycle, so the recommended optimal cycle number was set to 30 cycles.

#### 3.1.2 Annealing Temperature

Amplification specificity was determined by adjusting annealing temperature. Higher annealing temperature within the range of melting temperature could significantly reduce the non-specific binding between the primer and template. No locus dropout was detected in the annealing temperature between 56°C and 62°C (Supplementary Figure S1). Allele dropouts and an apparent decrease in peak height were observed at 64°C. Since better intra-color peak balance occurred at 60°C, it was determined to be the optimal annealing temperature.

#### 3.1.3 Final Extension Time

The final extension step aimed to ensure all double-stranded PCR products to be completely extended. The results demonstrated that the effect of varying final extension time by ±20 min from the optimal extension time of 20 min on the genotypes of the Y45 kit could be negligible. Generally, 20 min is the most recommended extension time (Supplementary Figure S2).

### 3.2 Sensitivity

**FIGURE 2:**
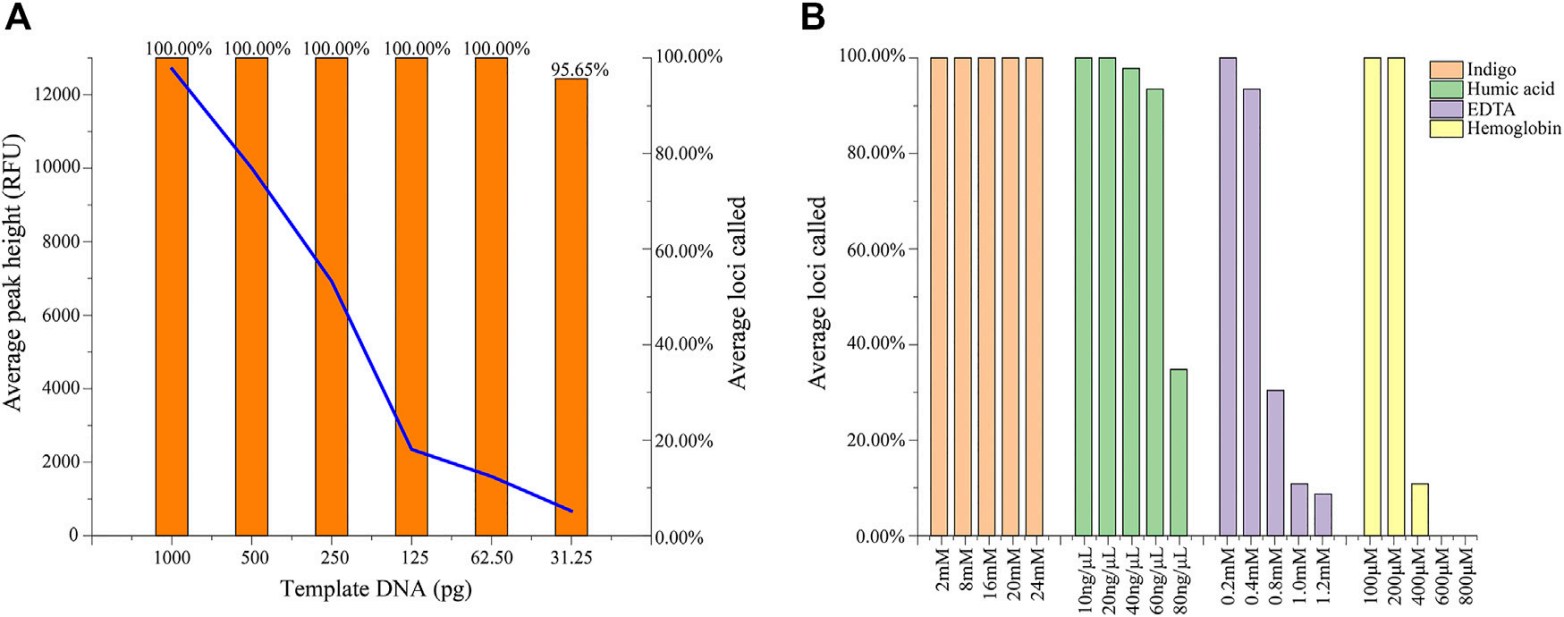
**(A)** Results of the sensitivity study based on the average peak height and loci called the ratio of control DNA 9948. **(B)** The average loci called ratio for four common inhibitors of indigo, humic acid, EDTA, and hemoglobin with different concentrations mixed with control DNA 9948.

Complete and accurate profiles were observed with DNA inputs ranging from 1 ng down to 61.5 pg by the threshold of 50 RFU. As shown in Figure 2A, when the DNA template was reduced to 31.25 pg, the average loci calling rate dropped to 95.65%, and dropouts at loci of DYS443 and Y_GATA_H4 were detected. Average peak height decreased from 12704 to 673 RFU with a reduction in the template concentration.

### 3.3 Inhibitor Study

Inhibitors that usually exist in the crime scene may affect the amplification of DNA samples and even cause failure. To evaluate the inhibitor tolerance of the Y45 kit, four common inhibitors including hemoglobin, indigo, humic acid, and EDTA were tested at verified concentrations. Complete profiles were obtained with 200 μM hemoglobin, 20 ng/μL humic acid, 0.2 mM EDTA, and all tested indigo concentrations (from 2 mM to 30 mM). When the concentration of humic acid was raised to 40 ng/μL, dropouts occurred in the loci with long fragment–sized alleles. With the concentration of hemoglobin exceeding 400 μM, allele dropouts were observed (Figure 2B).

### 3.4 Species Specificity

As expected, the primate DNA sample, chimpanzee, yielded a reproducible detectable product (Supplementary Figure S3), which scattered among loci DYS459, DYS437, DYS393, DYS392, DYS3891, DYS447, DYS439, and DYS622, and two peaks outside the marker range (OMR) were observed. For male pigs, the alleles at DYS456 and DYS459 were detected. Off-ladder peaks were also observed at DYS391, DYS392, and DYS456 from the male duck samples. While no artifacts were detected above 100 RFU for the other 5 non-human genomic samples. Therefore, the results demonstrated that the Y45 kit is of great specificity. However, circumspection should be paid when the samples are blended with the male primate, pig, or duck DNA.

### 3.5 Mixture Study

**FIGURE 3:**
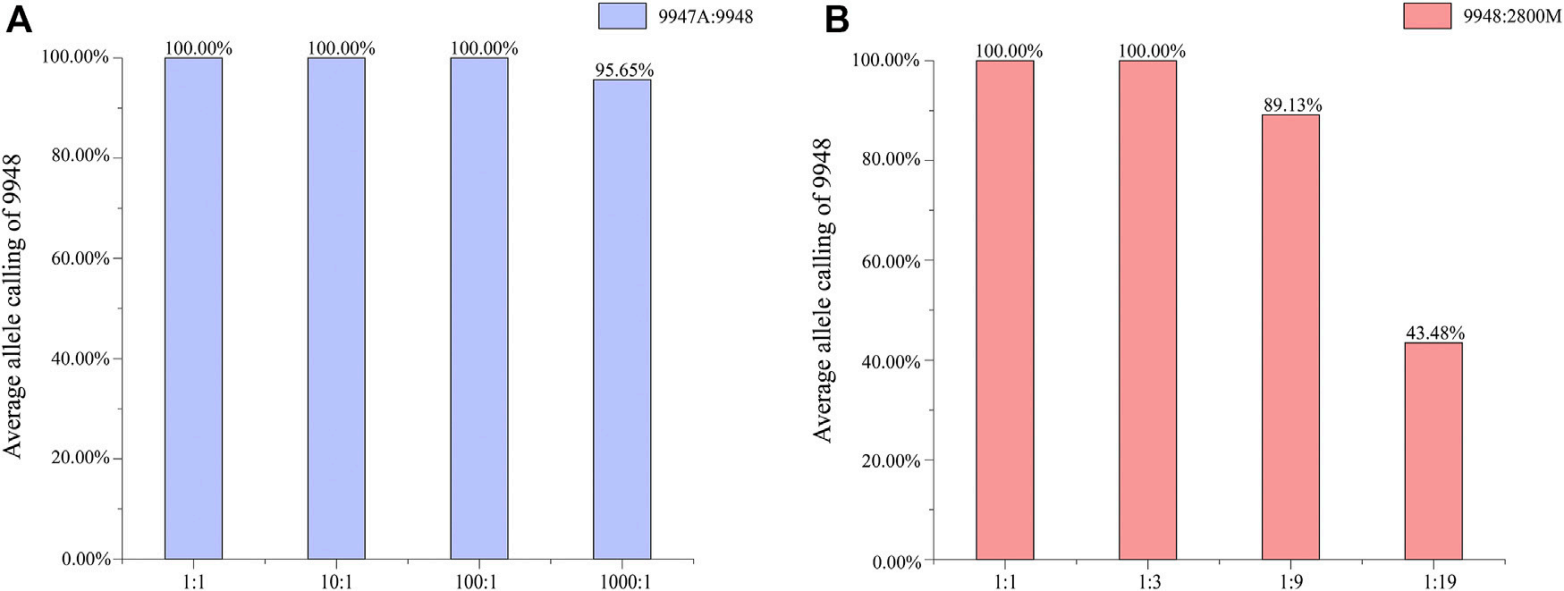
**(A)** Average allele calling ratio for different male/female mixtures. **(B)** The average allele calling ratio for different male/male mixtures.

Mixtures are commonly encountered in forensic casework. It is of high necessity to distinguish the major and minor components and figure out the contributor proportions. Female/male mixtures were prepared to access the amplification specificity and sensitivity of male samples in this system, while male/male DNA mixtures were used to evaluate the allele calling ratio of Y-STR typing of DNA samples at low concentrations in mixed samples. For the female/male mixtures, full profiles were obtained at ratios of 1:1, 10:1, and 100:1, and 95.65% alleles were called at 1,000:1 (Figure 3A). For the male/male mixtures (Figure 3B), a declined detectable rate of the minor male component 9948 was appeared accompanying the decreased ratios. All alleles were called at ratios of 1:1 and 1:3. Allele dropouts were detected in the 1:9 and 1:19 mixtures, resulting in an average of 89 and 43%, respectively.

### 3.6 Case Samples

A total of 30 samples consisting of ten case types were tested using the Y45 kit. The results showed that most of the case samples could generate complete DNA profiles, except for epitheliums on some samples. Full and accurate profiles were observed on muscle tissues, old bones, hair, nail, blood stains, semen, tooth, and saliva on cigarettes, while dropouts of some large size fragments were detected at DYS510 and DYS443 from DNA on the swabs of bottles and ropes (Supplementary Figure S4).

### 3.7 Reproducibility

Three laboratories were involved in the reproducibility study by genotyping the same samples. The results showed that the same samples got consistent genotypes among three participating laboratories, which demonstrated that profiles generated from the Y45 kit are reliable and suitable for comparison between laboratories (Supplementary Figure S5).

### 3.8 Stutter Calculation

Stutter is a by-product commonly generated in the amplification step owing to the slippage, which is usually one repeat unit shorter than the true allele. It may not seriously impact single sample typing but brings difficulties to mixed sample identification. The stutter ratios and relative parameters were calculated for 50 individuals from the population study. As presented in Supplementary Table S2, except for the loci of DYS481 (23.32%), DYS456 (15.45%), DYS389II (15.44%), and DYS393 (15.00%), the mean stutter ratios of other loci were lower than 15%. The recommended filter was determined by the mean plus or minus three standard deviations (SDs).

### 3.9 Population and Concordance Study

A total of 141 unrelated Han Chinese males were tested using the Y45 typing system, which was genotyped by the Yfiler Plus kit beforehand, and relevant allelic frequencies and forensic parameters were obtained. A total of 139 different haplotypes were generated, of which 137 haplotypes were unique, and 2 haplotypes were observed twice. The number of alleles at single-copy Y-STRs ranged from 2 for DYS645 to 11 for DYS557 (Supplementary Table S3). For multi-copy Y-STR loci (Supplementary Table S4), allelic combinations of DYS385a/b, DYS527a/b, and DYS459a/b were 48, 32, and 9, respectively. The values of HD, MP, and DC were 0.9998, 0.0073, and 0.9858, respectively. As shown in Supplementary Tables S3, S4, the gene diversity (GD) values of 45 Y-STR loci ranged from 0.1078 (DYS645) to 0.9666 (DYS385a/b).

## 4 Conclusion

Our study designed and developed a novel Y-STR system with increased loci number and high polymorphism in Han Chinese to satisfy the need for Y-STR database construction and forensic pedigree searches. A series of validation experiments were performed, and the results demonstrated that the Y45 kit is sensitive and robust, which demonstrated that the kit is capable of producing male-specific, reliable, and accurate profiles.

## Data Availability

The raw data supporting the conclusion of this article will be made available by the authors, without undue reservation.
