# Supplementary figures and images for: The Validation of a Single Multiplex Typing System With 45 Y-STR Markers for Familial Searching and Database Construction
Source: Front Genet. 2022 Jan 27;13:842004. doi: 10.3389/fgene.2022.842004 (PMC8829124; doi:10.3389/fgene.2022.842004)

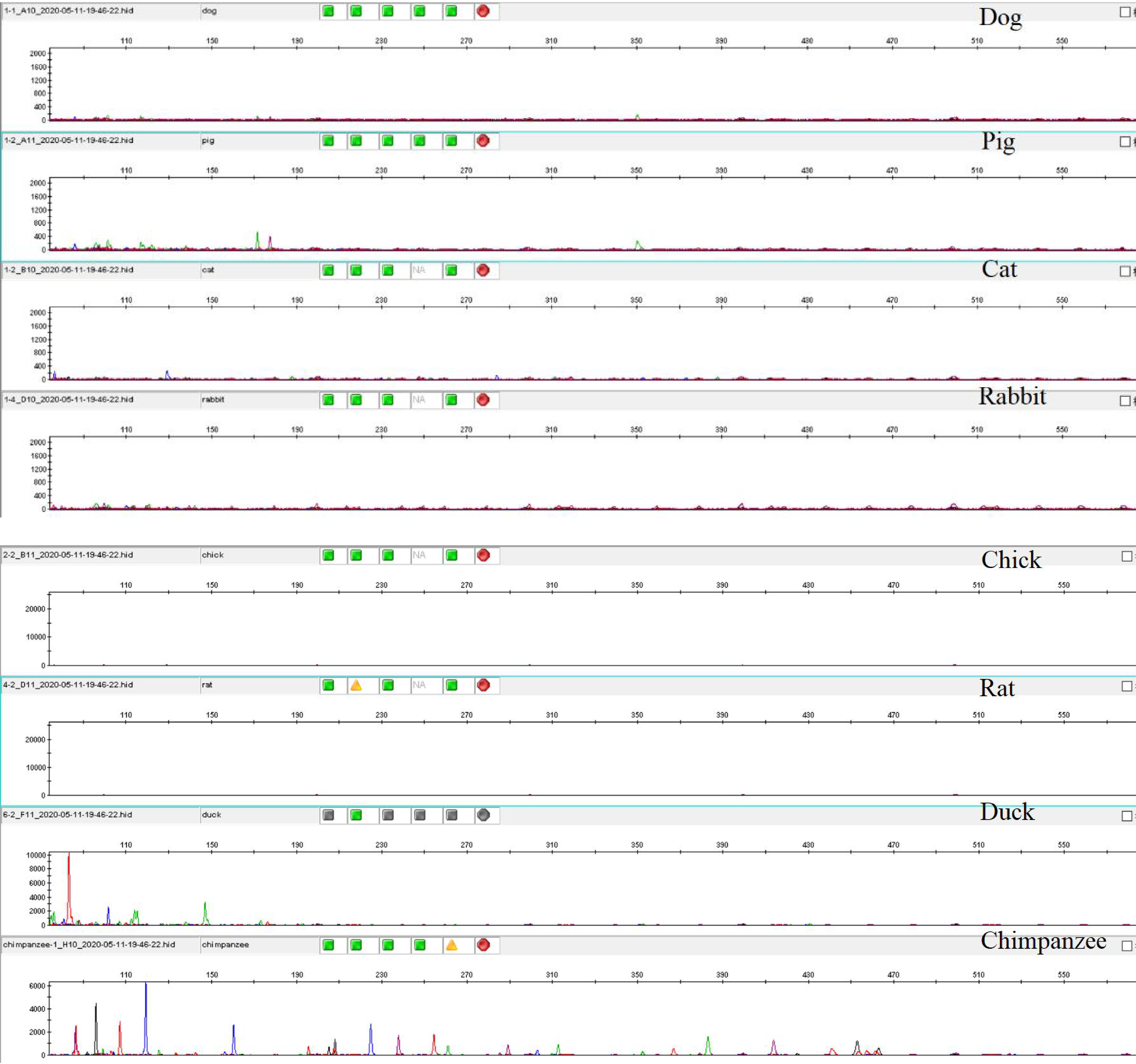

Supplement: Supplementary file 1 [file Image3.TIF]

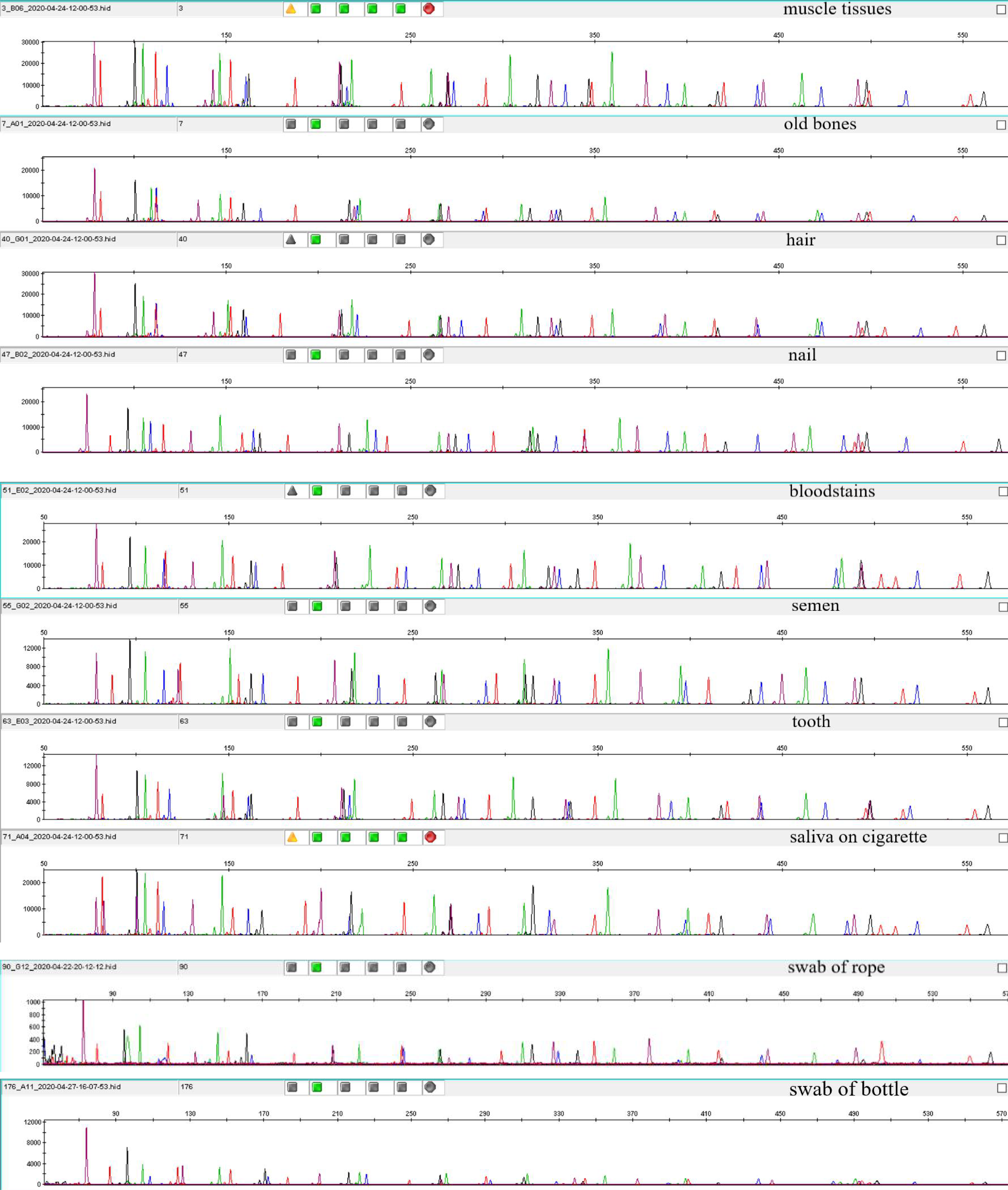

Supplement: Supplementary file 2 [file Image4.TIF]

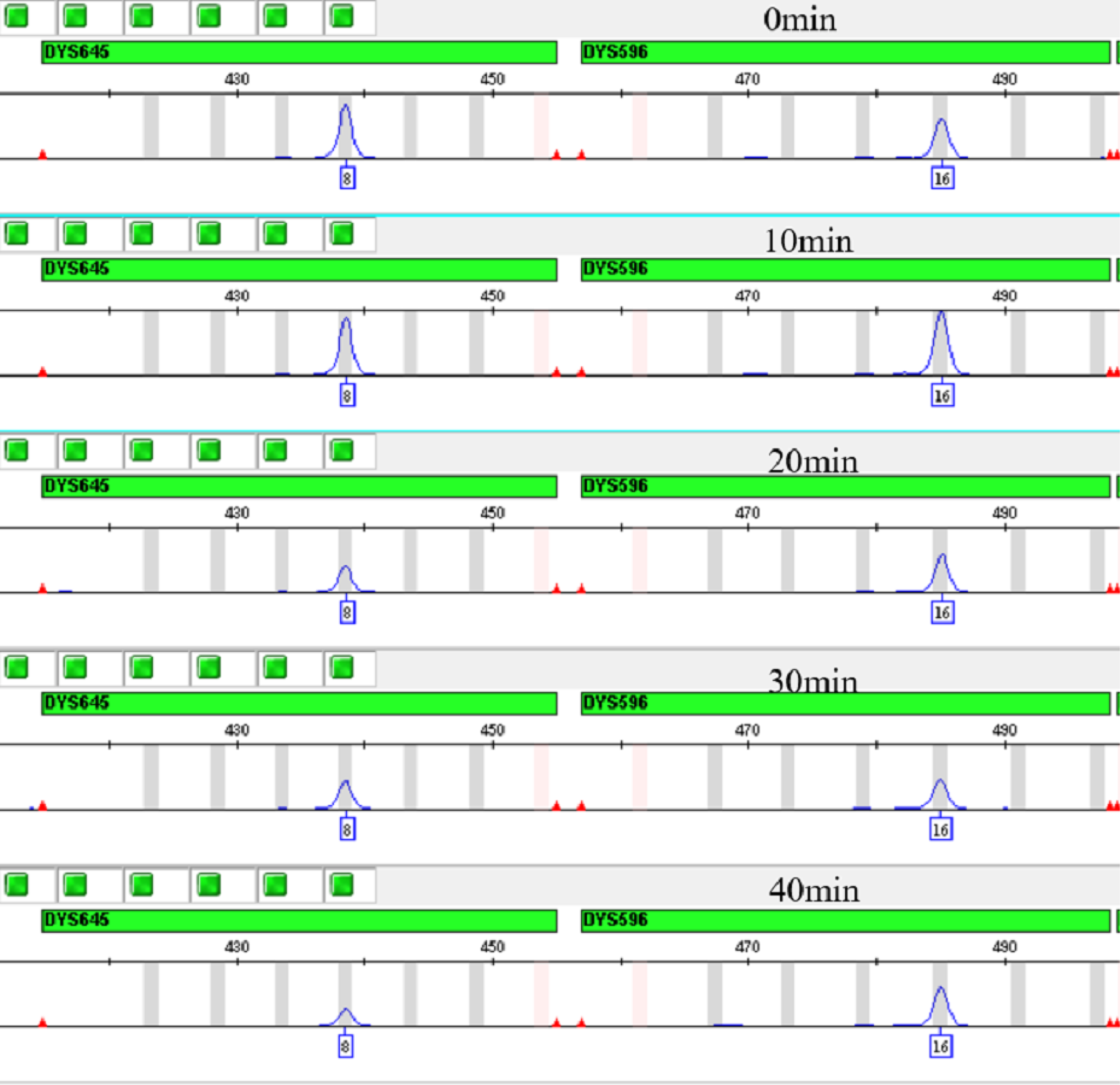

Supplement: Supplementary file 3 [file Image2.TIF]

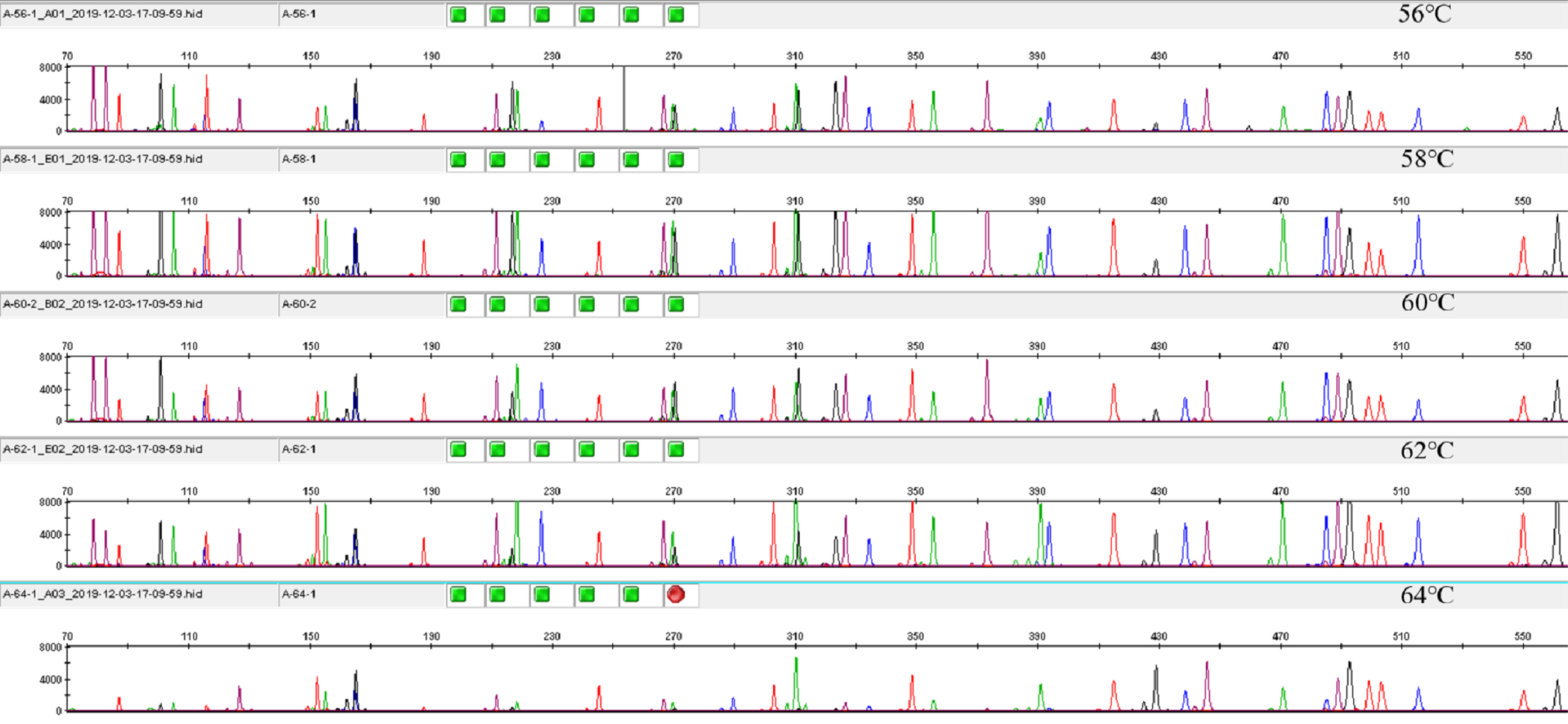

Supplement: Supplementary file 4 [file Image1.TIF]

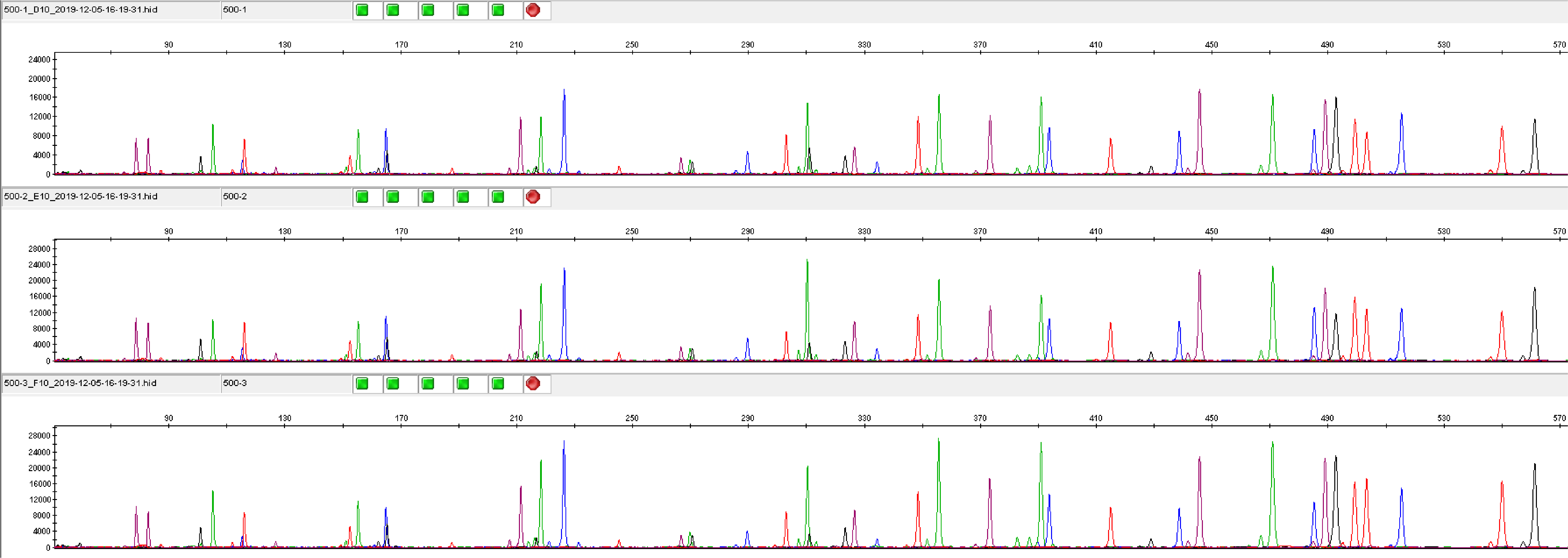

Supplement: Supplementary file 5 [file Image5.TIF]
